# Bibliometric analysis of IgA vasculitis nephritis in children from 2000 to 2022

**DOI:** 10.3389/fpubh.2022.1020231

**Published:** 2022-10-05

**Authors:** Fei Luo, Yuzhe Li, Yuan Zhang, Yehong Song, Juanjuan Diao

**Affiliations:** ^1^First College of Clinical Medicine, Shandong University of Traditional Chinese Medicine, Jinan, China; ^2^Department of Pediatrics, Affiliated Hospital of Shandong University of Traditional Chinese Medicine, Jinan, China; ^3^College of Traditional Chinese Medicine, Shandong University of Traditional Chinese Medicine, Jinan, China

**Keywords:** bibliometric analysis, IgA vasculitis nephritis, CiteSpace, VOSviewer, web of science

## Abstract

**Background:**

IgA vasculitis Nephritis (IgAVN) is a kidney-damaging disease that occurs during the course of IgA vasculitis (IgAV) and is the most serious complication of IgAV. However, there is a lack of reports of bibliometric analysis of IgAVN in children. The purpose of this study is to conduct a bibliometric analysis of IgAVN in children from 2000 to 2022, to explore the current status and cutting-edge trends in the field of IgAVN in children, and to establish new directions for subsequent research.

**Methods:**

Screening the literature in the field of IgAVN in children in the Web of Science Core Collection (WoSCC) from 2000 to 2022. Visual analysis of their annual publications, countries, institutions, authors, journals, keywords, and references were using CiteSpace5.8.R3 and VOSviewer1.6.18.

**Results:**

A total of 623 publications were included in the study, since the beginning of 2014, there has been an overall increasing trend in the number of articles issued. The most prolific country and institution were China and Zhejiang University. The most frequently cited author was Coppo R, with 331 citations, who has made great contributions to IgAVN. Mao Jianhua, Lee JS and Wyatt Robert J were the most prolific authors, all with 9 articles. Pediatric Nephrology was the most published and cited journal. The highest burst strength keyword is IgA vasculitis, and the highest burst strength reference is Davin JC, 2014.

**Conclusion:**

The research hotspots and trends predicted by the analysis of this study provide a reference for in-depth research in this field with a view to promoting the development of IgAVN research in children.

## Information

IgA vasculitis (IgAV), also known as Henoch-Schönlein purpura (HSP), is the most common autoimmune vasculitis in children, with a reported annual incidence of 3–27 cases per 100,000 children (1, 2). It is characterized by IgA-dominated immune deposits in the walls of small blood vessels throughout the body, which can involve the skin, kidneys, gastrointestinal tract and joints (3). The prognosis of IgAV is usually good, but renal damage is a major cause of morbidity and mortality (4, 5) and a major determinant of long-term prognosis (6). IgA vasculitis Nephritis (IgAVN) is a kidney-damaging disease that occurs during the course of IgAV and is one of the most serious complications of IgAV (7). IgAVN can manifest as microscopic or gross hematuria, protein-uria, nephrotic or nephritic syndrome, as well as acute renal failure (8). IgAVN is more common in children compared to adults and may progress to chronic kidney disease (CKD) or end-stage renal disease (ESRD) (9–11). Renal insufficiency has been reported in ~15% of children with IgAVN, which is only half the rate of adult IgAVN patients (12, 13).

Bibliometrics is a scientific method based on the literature system and bibliometric characteristics, applying mathematics, statistics and other measurement methods to analyze the distribution structure, quantitative relationships, and change patterns of literature (14). Bibliometric analysis is a statistical analysis and quantitative tool for research publications. Zhang et al. (15) analyzed the research trends of renal fibrosis in diabetic kidney disease from 1985 to 2020 by using CiteSpace. Ozbek et al. (16) used CiteSpace to visualize the most frequently cited pediatric brain tumors literature in the Web of Science database. Yu et al. (17) performed a visualization study on metabolomics in coronary artery disease by CiteSpace and VOSviewer. Web of Science (WOS), established in 1997, is largest comprehensive academic information resource covering the largest number of disciplines in the world, including the most influential core academic journals in various research fields. The literature on IgAVN in the WOS database is not small, but the overall literature is scattered, redundant, and lacking in effective sorting, and no bibliometric analysis of IgAVN in children has been reported. In order to ensure the quality and accessibility of data, we focus on the Web of Science Core Collection (WoSCC) database.

In this study, CiteSpace 5.8.R3 (18) and VOSviewer1.6.18 (19) were used to statistically analyze and visualize the literature related to IgAVN in children from 2000 to 2022. The main objectives of our study were to: (i) identify the major contributors to the field of IgAVN in children, including authors, institutions and countries; (ii) explore the current research status and future development trends; (iii) provide new directions for subsequent research on IgAVN in children.

## Materials and methods

We reviewed papers published in the past 20 years on WoSCC on April 28, 2022. Here are the search strategies: TS = (IgA vasculitis nephritis OR Henoch-Schönlein purpura nephritis OR purpura nephritis) AND TS = (Children OR Childhood OR Pediatric^*^).

Indexes = WoSCC, namely: Science Citation Index-Expanded (SCIE); The language was “English”, the document types included “article” and “review”. “Procedures paper”, “book chapter”, “data paper”, “early access” and “retracted publication” were excluded; publication time was from 2000/01/01 to 2022/4/28.

623 articles were screened out. The “fully recorded and cited references” of these documents were extracted into CiteSpace 5.8.R3 and VOSviewer1.6.18 in “plain text” format to identify the main countries, institutions, authors, journals, keywords and references.

## Result

### Annual numbers of publications

**Figure 1:**
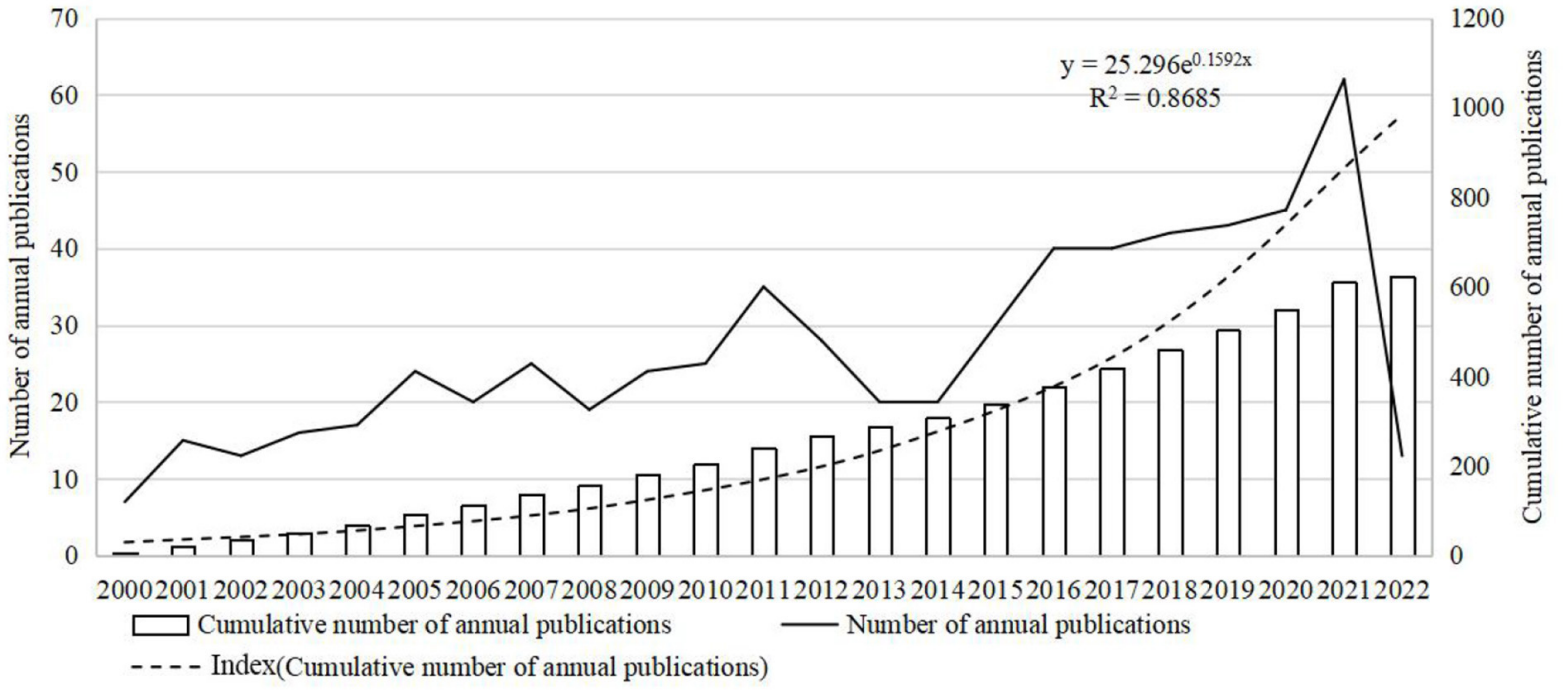
Trend of publications in the field of IgAVN in children (2000–2022).

From 2000 to 2022, WoSCC published 623 publications about IgAVN in children, of which 543 articles and 80 reviews. As shown in Figure 1, the number of annual publications of pediatric IgAVN has fluctuated slightly over the past 22 years. During this period, although the number of publications decreased, it showed an overall upward trend, indicating that IgAVN in children has been widely concerned with the medical progress. From 2000 to 2005, from 2008 to 2011 and from 2014 to 2021, the number of publications issued showed an increasing trend. However, the number of articles published decreased from 35 in 2011 to 20 in 2013. The number of articles published in 2014 stabilized at 20 and increased to 30 in 2015. The lowest number of documents was in 2000 (*n* = 7), and reached the peak in 2021 (*n* = 62). Although the number of publications decreased during the period, it showed an overall upward trend, indicating that children's IgAVN has received extensive attention with the progress of medicine.

### Analysis of countries and institutions

**Figure 2:**
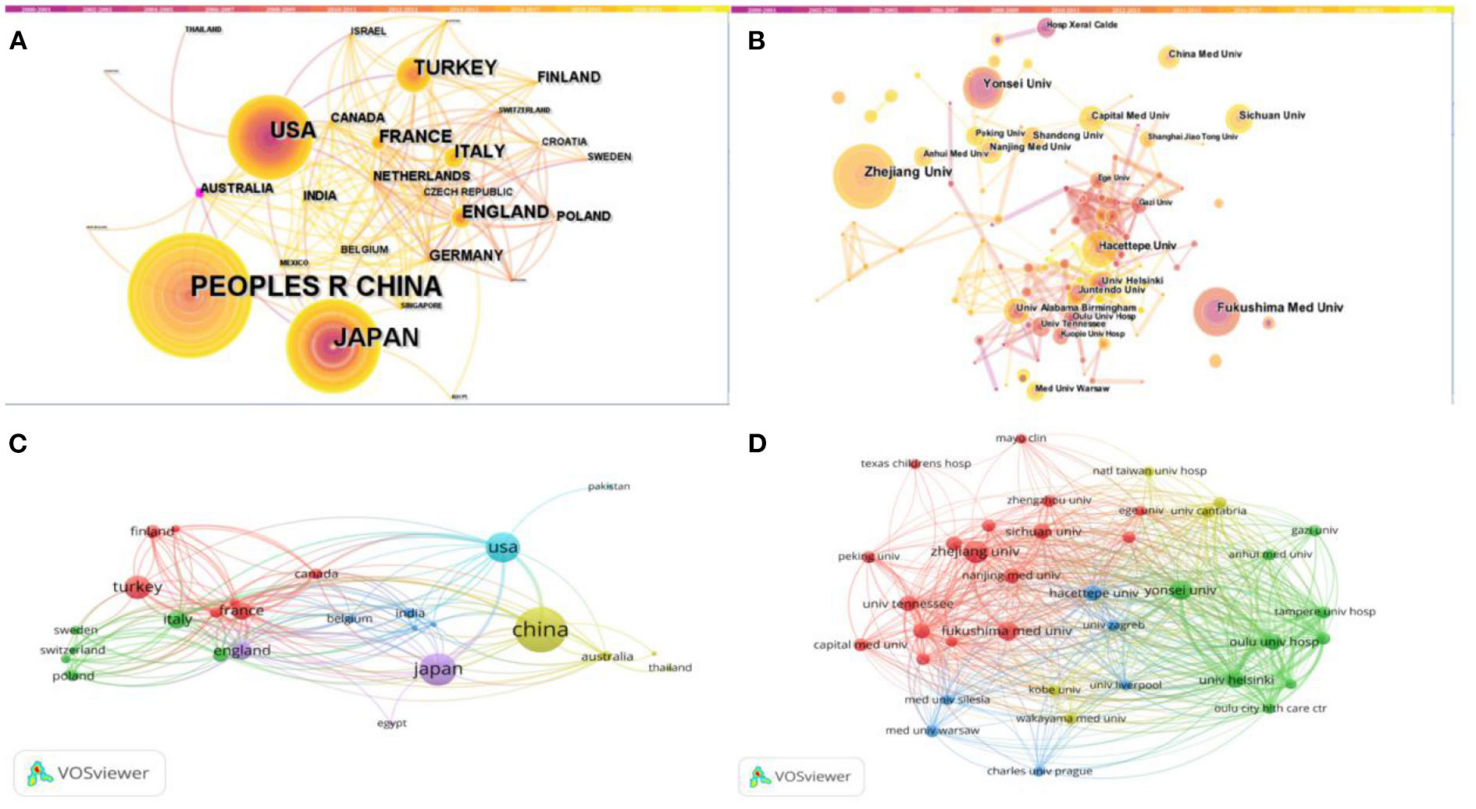
Analysis of the countries and institutions. **(A)** CiteSpace network map of countries involved in IgAVN in Children. **(B)** CiteSpace network map of institutions involved in IgAVN in Children. **(C)** VOSviewer network map of countries involved in IgAVN in Children. **(D)** VOSviewer network map of institutions involved in IgAVN in Children. Peoples r china and taiwan were replaced by China; united states was replaced by USA; north ireland, wales, england and scotland were replaced by England.

CiteSpace and VOSviewer were used to map cooperation between countries (Figures 2A,C) and institutions (Figures 2B,D). Tables 1, 2 lists the top 5 countries and institutions. In the field of IgAVN in Children, China made the largest contribution (*n* = 182, 29.2%), followed by Japan, the USA, Turkey and South Korea. Institutions with the largest number of papers is Zhejiang University (China), followed by Fukushima Medical University (Japan). As shown in Figures 2A,B, Australia and University Alabama Birmingham are marked by purple circles and have high centrality, indicating that they promote the research progress in the field of IgAVN in Children. As shown in Figure 2C, different colors represent intimate relationship clusters, and countries with close cooperation can be subdivided into 5 types. The blue part shows that the USA has more cooperation with Pakistan, India and Belgium; the yellow part shows that China has close ties with the USA and has cooperation with Australia and England; the green part, Italy frequently cooperates with Sweden, Poland, Switzerland and Germany; the red part, Turkey has more cooperation with Finland, Canada and France; the purple part, England has close ties with Japan and Egypt. As shown in Figure 2D, the red part shows that Zhejiang University frequently cooperates with Sichuan University, University Tennessee and Fukushima Medical University; the green part, Yonsei University and University Helsinki cooperate more closely; the yellow part shows that University Cantabria cooperates closely with Complejo Hospitalio Xeral Calde; the blue part, Hecettepe University is closely related to University Zagreb.

**Table 1:** The top 5 most publication countries.

**Rank**	**Countries**	**Count (%)**	**Countries**	**Centrality**
1	China	182 (29.2%)	Australia	0.1
2	Japan	93 (14.9%)	England	0.08
3	USA	77 (12.4%)	USA	0.07
4	Turkey	47 (7.5%)	France	0.05
5	South Korea	32 (5.1%)	Italy	0.04

**Table 2:** The top 5 most publication institutions.

**Rank**	**Institutions**	**Count (%)**	**Institutions**	**Centrality**
1	Zhejiang Univ	20 (3.2%)	Univ Alabama Birmingham	0.09
2	Fukushima Med Univ	15 (2.4%)	Univ Toronto	0.08
3	Yonsei Univ	13 (2.0%)	Hacettepe Univ	0.07
4	Hacettepe Univ	11 (1.8%)	Juntendo Univ	0.06
5	Sichuan Univ	9 (1.4%)	Univ Michigan	0.04

### Analysis of authors and cited authors

**Figure 3:**
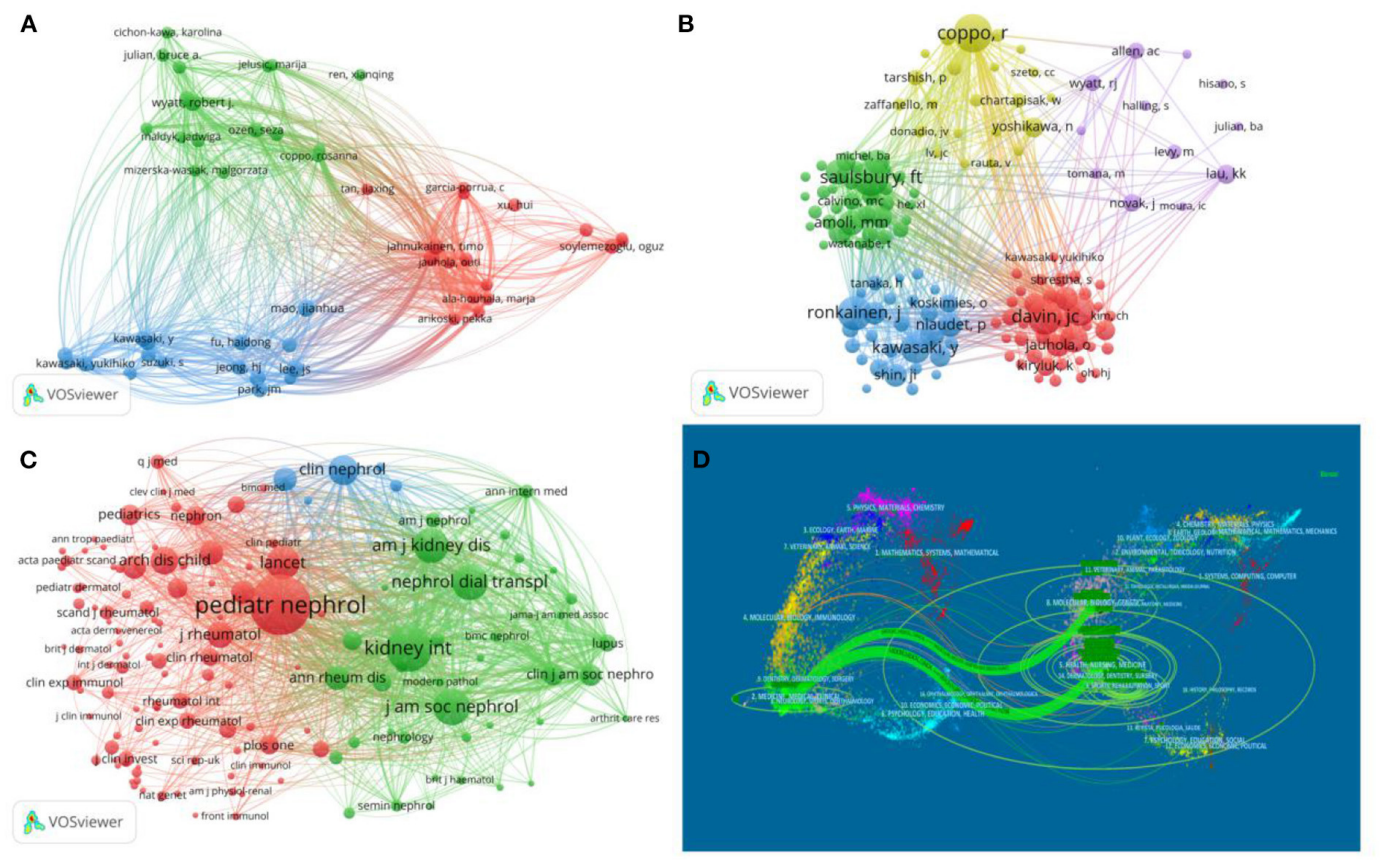
Analysis of the authors and journals. **(A)** Visualization of authors. **(B)** Visualization of cited authors. **(C)** Visualization of cited journals. **(D)** The dual-map overlay of IgAVN in Children research.

The top 5 authors of IgAVN in Children are shown in Table 3, with Mao Jianhua from Zhejiang University, Lee JS of Yansi University and Wyatt Robert J of the University of Tennessee tied for the first place, with 9 articles all published. As shown in Figure 3A, different colors represent closely cooperative clusters. Mao Jianhua, Lee JS, Wyatt Robert J, Fu haidong and Jahnukainen Timo are in different clusters, but they cooperate with each other. The top 5 most frequently cited authors are from North America and Europe (Table 4). The top 3 cited authors, Coppo R(331), Saulsbury FT(287) and Davin JC(281), belonging to different cooperative groups and occupying the core position (Figure 3B).

**Table 3:** The top 5 most publication authors.

**Rank**	**Author**	**Publications**	**Country**	**Institution**
1	Mao, Jianhua	9	China	Zhejiang University
2	Lee, JS	9	South Korea	Yonsei University
3	Wyatt, Robert J	9	USA	University of Tennessee
4	Fu, Haidong	8	China	Zhejiang University
5	Jahnukainen, Timo	8	Finland	University of Helsinki

**Table 4:** The top 5 most publication cited authors.

**Rank**	**Cited**	**Cited**	**Country**	**Institution**
	**author**	**frequency**		
1	Coppo, R	331	Italy	Regina Margherita Hospital
2	Saulsbury, FT	287	USA	University Virginia
3	Davin, JC	281	Netherlands	University Amsterdam
4	Ronkainen, J	264	Finland	University Oulu
5	Ozen, S	217	Turkey	Hacettepe University

### Analysis of journals and cited journals

As shown in Table 5, Pediatric Nephrology (IF 3.65) is the most prolific journal, with a total of 83 papers published. Nephrology Dialysis Transplantation, Clinical Nephrology, Clinical Rheumatology and Pediatric Rheumatology rank second, both with 12 papers published. Besides, Pediatric Nephrology is also the most cited journal (Table 6). In the journal co-occurrence map (Figure 3C), Pediatric Nephrology is the largest node. It can be seen that Pediatric Nephrology is the core journal with the largest number of papers in the field of children's IgAVN, indicating that its comprehensive strength and influence are better than other journals. The double graph overlay of journals (Figure 3D) shows the relationship distribution among journals, with the citing journals on the left and the cited journals on the right (20, 21). The green path indicates that papers published on Molecular/Biology/Genetics/Health/Medicine/Nursing are often cited in the Medical/Medicine/Clinical issue.

**Table 5:** The top 5 most publication journals.

**Rank**	**Journal**	**Publications (%)**	**IF**	**JCR**
			**(JCR 2021)**	**quartile**
1	Pediatric Nephrology	83 (13.3%)	3.65	Q2
2	Nephrology Dialysis Transplantation	12 (1.9%)	7.19	Q1
3	Clinical Nephrology	12 (1.9%)	1.24	Q4
4	Clinical Rheumatology	12 (1.9%)	3.65	Q3
5	Pediatric Rheumatology	12 (1.9%)	3.41	Q2

**Table 6:** The top 5 most publication cited journals.

**Rank**	**Cited**	**Citations**	**IF**	**JCR**
	**journal**		**(JCR 2021)**	**quartile**
1	Pediatric Nephrology	1983	3.65	Q2
2	Kidney International	1116	19	Q1
3	Journal of The American Society of Nephrology	719	9.27	Q1
4	Nephrology Dialysis Transplantation	695	7.19	Q1
5	American Journal of Kidney Diseases	655	11.07	Q1

### Analysis of keywords

As shown in Table 7, the top 10 keywords with the highest frequency in the past 20 years are children(363), henoch-schöenlein purpura (279), nephritis (264), iga nephropathy (160), disease (120), childhood (103), adult (99), henoch-schönlein purpura nephritis (99), vasculitis (86), therapy (80).

**Table 7:** Top 20 keywords with the highest frequency of occurrence.

**Rank**	**Keyword**	**Occurrences**	**Rank**	**Keyword**	**Occurrences**
1	Children	363	11	Glomerulonephritis	78
2	Henoch-Schönlein purpura	279	12	Prognosis	77
3	Nephritis	264	13	IgA vasculitis	71
4	IgA nephropathy	160	14	Classification	69
5	Disease	120	15	Renal involvement	60
6	Childhood	103	16	Nephropathy	55
7	Adult	99	17	Outcome	48
8	Henoch-Schönlein purpura nephritis	99	18	Follow-up	46
9	Vasculitis	86	19	Proteinuria	44
10	Therapy	80	20	Long-term prognosis	43

**Figure 4:**
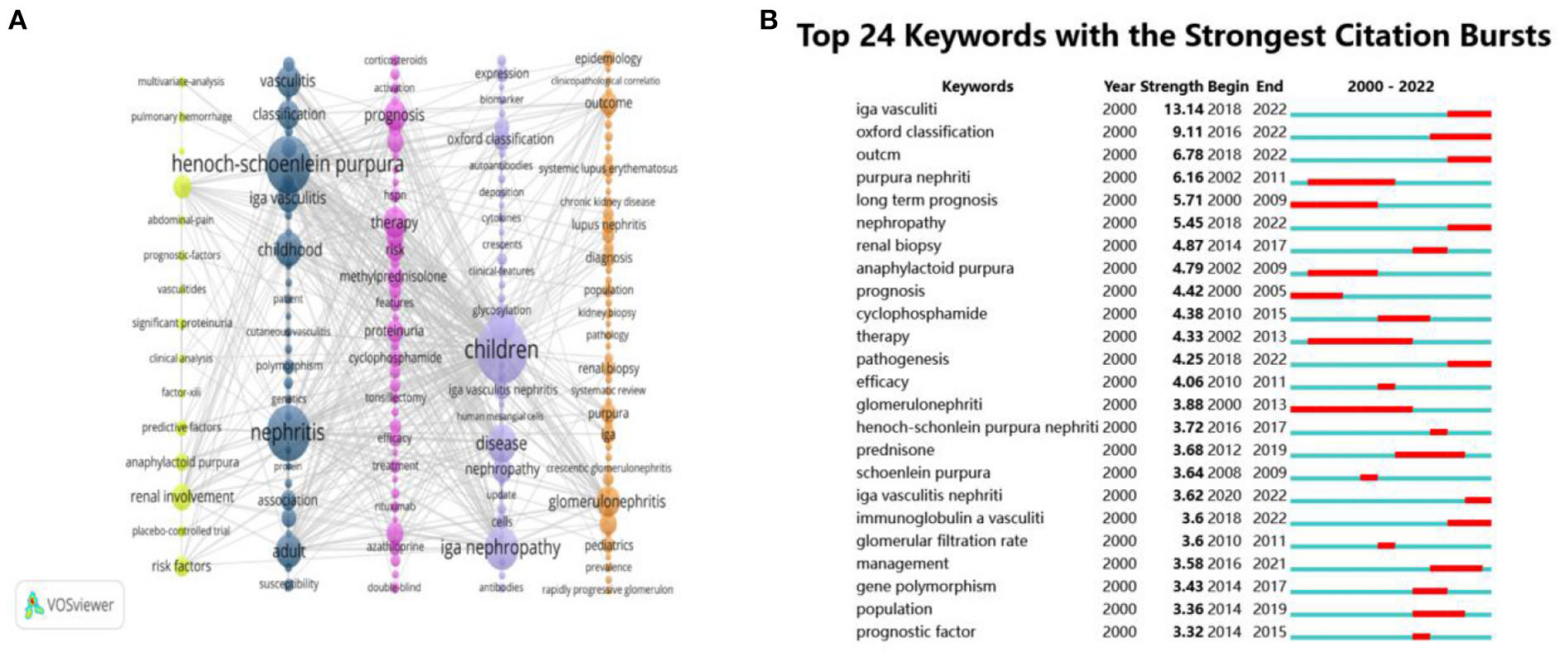
The analysis of keywords. **(A)** Visualization of keywords. **(B)** The top 24 keywords with the strongest citation bursts.

As shown in Figure 4A, it can be divided into 5 clusters according to color. The yellow part: follow-up, renal involvement, risk factors; the black part: classification, henoch-schönlein purpura, iga vasculitis, childhood, nephritis, association, adult; the pink part: prognosis, purpura nephritis, therapy, risk, methylprednisolone, proteinuria; the purple part: children, oxford classification, disease, iga nephropathy; the orange part: outcome, lupus nephritis, glomerulonephritis, renal biopsy, nephrotic syndrome.

Through the burst detection analysis of keywords, 24 co-occurrence words were obtained. The results are shown in Figure 4B. Long term prognosis, prognosis and glomerulonephritis are the keywords of the earliest outbreak. Glomerular filtration rate, prednisone and renal biopsy broke out from 2010 to 2014. Oxford classification, management and pathology broke out from 2016 to 2018. The lastest is igA vasculitis. The keyword with the highest strength is IgA vasculitis, with a score of 13.14, the second is Oxford classification, with a score of 9.11.

### Analysis of references

**Figure 5:**
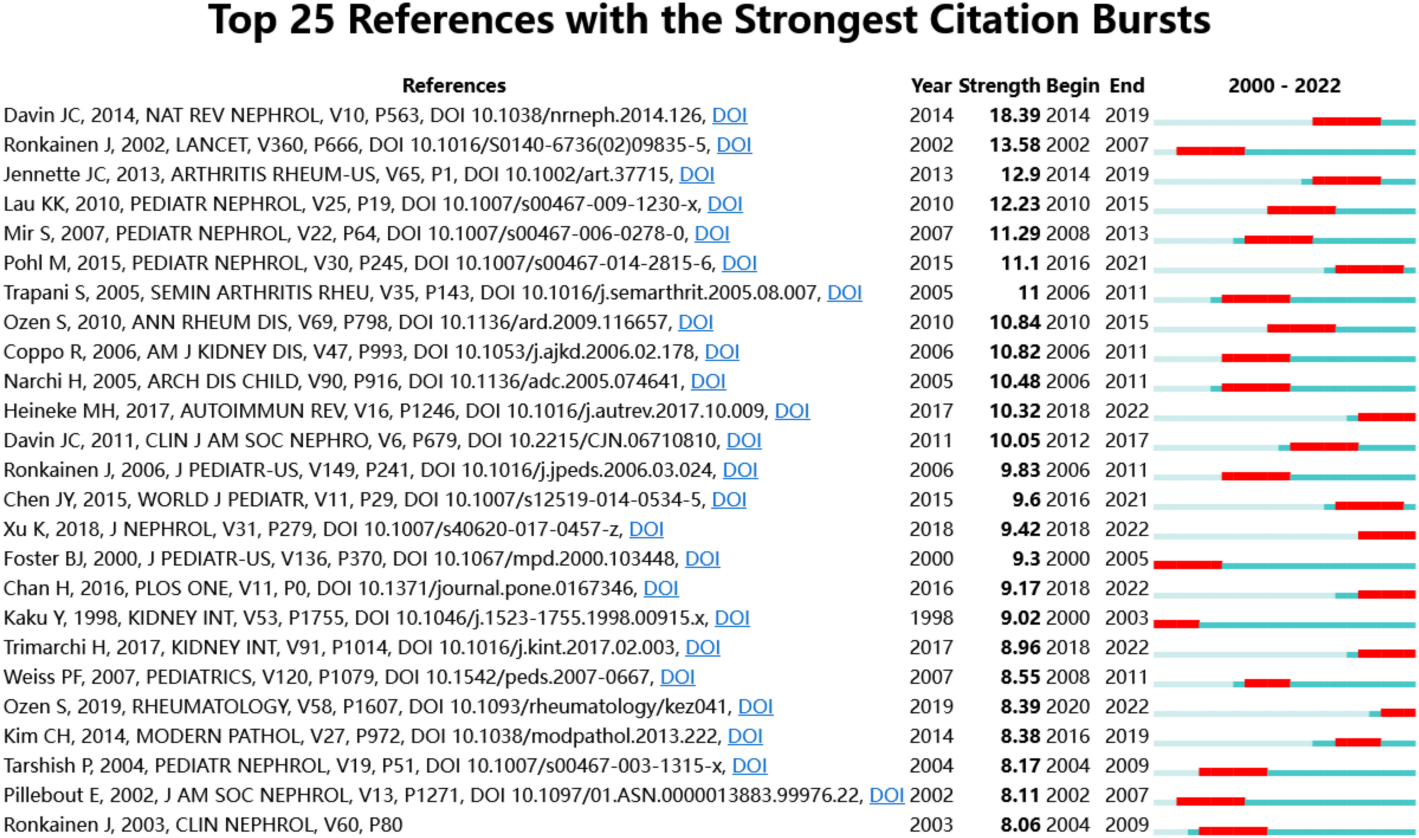
The top 25 cited references with the strongest citation bursts.

The Supplementary Table 1 lists the 10 most frequently cited references. The most frequently cited one was published by Goldstein et al. (22) in Lancet in 1992, followed by Gardner-Medwin et al. (23) in Lancet in 2002. Based on the burst detection analysisthe references, the results of the first 25 articles are shown in Figure 5. The article with the highest burst strength was published on Nature reviews Nephrology by Davin in 2014 (18.39), and the second was published by Ronkainen et al. in Lancet in 2002 (13.58).

## Discussion

IgAVN is the most common secondary glomerular disease in children and one of the main causes of ESKD (11). The disease is repeated and difficult to heal, which also seriously affects the quality of life of children with IgAVN. Therefore, it is of great significance to actively prevent IgAVN.

The study was conducted by using CiteSpace5.8.R3 and VOSviewer1.6.18 search of 623 IgAVN in children publications published between 2000 and 2022 in the WOS database for statistical analysis and visualization. The paper analyzes the current situation and trend of annual publications, countries, institutions, authors, periodicals and research.

In recent two decades, IgAVN related publications have generally shown an upward trend, the number of publications increased significantly from 2016 to 2021, and the number of publications in 2021 was 9 times that in 2000. It reveals that this field has attracted more and more scholars' attention in recent years.

In terms of countries and institutions, China, Japan and the USA are the main research countries of IgAVN in children. Centrality value >0.1 is a relatively significant node. As can be seen from Figures 2A,B, the distribution of countries and institutions is scattered, and the centrality of each institution is <0.1, indicating that there is less international cooperation and academic exchanges. China is the country with the largest number of publications, and 2 of the top 5 institutions are located in China, but the country and institution with the highest centrality is not China, indicating that China does not have strong cooperation with other countries. On the contrary, Australia, which does not have many publications, is the country with the highest centrality and the closest cooperation.

As far as authors are concerned, Mao Jianhua from Zhejiang University, Lee JS from Yonsei University and Wyatt Robert J from Tennessee University are tied for the first place, and they are the most prolific authors. Mao's research involves the pathogenesis and clinical manifestation of IgAVN in children (24–26), Lee mainly studies the treatment and prognosis of IgAVN in children (27–29), Wyatt mainly discusses the mechanism of IgA1 in IgAVN in children (30–32). Coppo R is the most frequently cited author, with 331 citations. She is mainly involved in the formulation of evidence-based guidelines for IgAVN (33). In addition, she has worked closely with experts from various cooperative groups and made great contributions to the study of IgAVN.

Pediatric Nephrology (IF 3.65 Q2) was the most prolific and cited journal, with a total of 83 articles, indicating that the journal has a strong influence in the field of pediatric IgAVN research and is favored by the majority of scholars. Among the top 5 journals, only one journal has IF > 5, indicating that the quality of research papers in this field needs to be improved. Most of the journals in Table 6 are from Q1 or Q2, which reveals that the research of IgAVN has been highly valued by scholars all over the world.

From the analysis of references, the most frequently cited papers were published by Goldstein AR. This study reveals that children with IgAVN need long-term follow-up, especially pregnant women (22). The paper with the highest burst strength was published by Davin JC, followed by Ronkainen J. Davin et al. (34) found that the long-term prognosis of IgAVN mainly depends on the development of CKD, but the early clinical and histological manifestations can not predict the risk of CKD. CKD can be observed during long-term follow-up even after IgAVN is fully recovered. Dudley et al. (35) showed that the early use of prednisolone in the treatment of IgAV could not prove that it could reduce the incidence of proteinuria in sick children 12 months after onset.

According to keyword co-occurrence, clustering and burst detection analysis, two research priorities in the field of IgAVN in children are determined: pathogenesis and management. (i) pathogenesis of IgAVN in children: At present, it is generally recognized that the “multiple blows” theory (36, 37). Galactose-deficient IgA1 synthesis (Step1), IgG antibody response to Gd-IgA1 (Step2), formation of pathogenic Gd-IgA1 immune complexes (Step3), and thylakoid deposition of these immune complexes leads to renal inflammation through complement system activation (Step4). In short, abnormal glycosylated IgA1 is deposited in the glomerular mesangial region, which induces mesangial cell proliferation, activation and release of various inflammatory mediators, resulting in glomerular injury. The disease most associated with IgAVN is IgA Nephrology (IgAN). Both IgAN and IgAVN have the pathogenesis dominated by galactose-deficient IgA1 (Gd-IgA1). Suzuki et al. (38) found that Gd-IgA1 can be detected in IgAN and IgAVN. It is reported that aberrant glycosylation of IgA1 is inherited in both IgAN and IgAVN in children (30). Torun (39) and Sanders (40) believe that IgAVN children and IgAN children have similarities in clinical and histology, and are closely related. Long-term renal outcome was good in both IgAVN and IgAN. (ii) management of IgAVN in children: The Kidney Disease Improving Global Outcomes (KDIGO) suggests that IgAVN can be treated similar to IgAN (41). For children with severe IgAVN, KDIGO and European treatment guidelines recommend cyclophosphamide (CYC) therapy and steroid immunosuppressive therapy. For children with mild or moderate IgAVN, KDIGO recommends immunosuppressive drugs, while European treatment guidelines recommend glucocorticoid therapy. Oxford classification is also one of the hotspots in recent years. At present, International Study Group of Kidney Disease in Children (ISKDC) classification is mainly used (42), (I)minimal glomerular alterations; (II) mesangial proliferation; (IIIa) focal or (IIIb) diffuse pro-liferation or sclerosis with <50% crescents; (IV) mesan-gial proliferation or sclerosis with 50–75% crescents; (V) mesangial proliferation or sclerosis with >75% crescents; (VI) membranoproliferative-like lesion. ISKDC predicts renal prognosis mainly based on the degree of crescent formation. Some scholars believe that ISKDC lacks consensus on the value of crescent as a long-term prediction index, and there are some limitations. When immunosuppressive therapy is used, the predictive value of the crescent is of little significance (43). The Oxford Classification for IgAN includes mesangial proliferation (M), endocapillary hypercellularity (E), segmental glomerulosclerosis (S), tubular atrophy/interstitial fibrosis (T), and cellular/fibrocellular crescents (C). The researchers pointed out that the updated Oxford classification is applicable to IgAVN, both in children and adults, and may also aid in disease management and kidney outcome prediction of IgAVN (3, 44). Wang et al. (45) found that the S and T of Oxford classification can be used to predict the renal prognosis of IgAVN children.

It is worth noting that this paper still has some limitations. First, this study only searched publications from WoSCC, which may be incomplete. Secondly, the choice of language (English) and literature type (article and review) may lead to deviation in the results. Thirdly, the limitations of software may also lead to mistakes.

## Conclusion

Through bibliometric methods, this study makes a visual analysis of papers in the field of IgAVN in children from 2000 to 2022. Although the number of literature in this field is limited, it shows an upward trend as a whole, the publications have increased significantly in recent years, indicating that more and more scholars pay more attention to IgAVN in children. In short, the study provide assistance to scholars seeking academic collaboration, new ideas for clinicians and researchers, and new guidance for the development of the field.

## Data availability statement

The raw data supporting the conclusions of this article will be made available by the authors, without undue reservation.

## Author contributions

JD put forward the idea of the article and supervised the study. Software and the figures were provided by YL, YZ, and YS carried out the data preparation and network construction. FL analyzed the result and wrote the manuscript. JD participated and directed the revision of the manuscript. All authors read and approved the final version of the manuscript.

## Conflict of interest

The authors declare that the research was conducted in the absence of any commercial or financial relationships that could be construed as a potential conflict of interest.

## Publisher's note

All claims expressed in this article are solely those of the authors and do not necessarily represent those of their affiliated organizations, or those of the publisher, the editors and the reviewers. Any product that may be evaluated in this article, or claim that may be made by its manufacturer, is not guaranteed or endorsed by the publisher.
